# Portfolio Selection with Irregular Time Grids: an example using an ICA-COGARCH(1, 1) approach

**DOI:** 10.1007/s11408-021-00387-3

**Published:** 2021-03-31

**Authors:** Francesco Bianchi, Lorenzo Mercuri, Edit Rroji

**Affiliations:** 1 Independent, Durham, Italy; 2 University of Milan, Milan, Italy; 3 CREST Japan Science and Technology Agency, Tokyo, Japan; 4 University of Milano-Bicocca, Milan, Italy

**Keywords:** Irregular grids, Independent Component Analysis, Continuous GARCH, Risk measures, C51, C11, G17

## Abstract

In this paper we consider a portfolio selection problem defined for irregularly spaced observations. We use the Independent Component Analysis for the identification of the dependence structure and continuous-time GARCH models for the marginals. We discuss both estimation and simulation of market prices in a context where the time grid of price quotations differs across assets. We present an empirical analysis of the proposed approach using two high-frequency datasets that provides better out-of-sample results than competing portfolio strategies except for the case of severe market conditions with frequent rebalancements.

## Introduction

Conditional heteroskedasticity is a well-known stylized fact observed in financial time series. Generalized Autoregressive Conditional Heteroskedastic (GARCH) models have been widely used since their introduction in Bollerslev (1987). The main drawback of GARCH models, defined in discrete time, is that they assume realizations are collected on an equally spaced time grid, but if we consider, for example, daily data some irregularities appear due to market closing, weekends or bank holidays. The same holds true in high-frequency trading as market activity in small time intervals depends on the liquidity of the asset.

The Continuous GARCH($p,\,q$) model, namely COGARCH($p,\,q$), introduced in Brockwell et al. (2006) shares many similarities with the GARCH model. Indeed, the same noise process drives both the observable and the variance process; the variance in the COGARCH($p,\,q$) model is a Continuous ARMA (CARMA) model (see Brockwell and Davis 2016). Differences emerge in the estimation of the two models. Recently, Iacus et al. (2018) proposed a discrete time process that is shown to converge to the COGARCH($p,\,q$) model. The innovations in the approximating process are constructed using the first jump approximation method (see Maller and Szimayer 2007), while model parameters are estimated through the pseudo-maximum likelihood method.

A challenging task in portfolio selection is the modeling of the joint behavior of asset prices. Standard approaches are based on parametric multivariate distributions, while alternative approaches build on the possibility of separating the task of dependence structure identification from the study of marginal features. For instance, we can use copula functions (see Babaei et al. 2015, and references therein) or blind source separation (see Acharyya 2008, for details). The latter is based on the assumption that observable series are a combination of unobservable signals. Extraction of signals can be performed for example by means of the Independent Component Analysis (ICA) introduced in Comon (1994) that considers the observed series as a linear combination of independent non-Gaussian signals. Starting from Back and Weigend (1998), several authors have applied the ICA in forecasting financial time series for example in Lu et al. (2009) or for portfolio selection (see Madan and Yen 2004; Madan 2006). In Hitaj et al. (2015) some parametric and nonparametric distributions are considered for the independent components in a portfolio optimization problem for a Constant Absolute Risk Aversion (CARA) utility function.

The ICA algorithm has been also employed for the construction of multivariate GARCH($p,\,q$) processes, called ICA-GARCH($p,\,q$) (see Broda et al. 2013, for instance) and in portfolio optimization (see Chen et al. 2007, for optimal portfolios where the risk measure in the objective function is the Value at Risk). The ICA-GARCH($p,\,q$) model assumes data are equally spaced, i.e., no effect is assigned to missing data. Moreover, if we have data observed on a regularly space time grid, for instance daily data, we cannot generate future scenarios with a higher time frequency, e.g., intraday data or with a lower frequency that is not a multiple of the time distance between two observations.

We present a framework based on the ICA-COGARCH(1, 1) model where the independent non-Gaussian signals are assumed to follow a COGARCH(1, 1) process. In the literature, a similar approach in discrete-time context has been proposed in Broda and Paolella (2009) where the extracted factors are assumed to be described through GARCH(1, 1) models.^1^ As in our framework, it is based on a two-step procedure where the estimation of the correlation structure precedes the univariate modeling of extracted components. The main difference with our proposal is that Broda and Paolella (2009) exploit the time structure of the dataset to identify the independent components. In particular, the independent components are extracted by maximizing the autocorrelation of squared returns but with additional parameter restrictions as the procedure requires finiteness of the fourth moments of all observed financial time series. We apply directly the FastICA algorithm in Hyvarinen and Oja (1997) to the series of returns without considering the autocorrelation structure of data, but we do not make any assumption on the higher moments of asset returns.

The objective function in the portfolio selection problem is a combination of the expected terminal wealth and a specific risk measure^2^. Another possible way to address this modeling issue would be to use multivariate COGARCH processes as defined in Stelzer (2010) but with additional numerical estimation burden in a multivariate context where the fitting is based on a quasi-maximum likelihood procedure. We propose a less complex framework and present an empirical analysis of the proposed approach using two high-frequency datasets composed of the members of the FTSE 100 Index.

Results of the portfolio selection problem are presented for different risk aversion profiles. We discuss the results of a rolling window strategy both from an in-sample and from the out-of-sample point of view. The proposed approach provides better out-of-sample results than competing portfolio strategies except for the case of financial turmoils as observed recently during the COVID19-induced crisis.

The outline of the paper is as follows. In Sect. 2 we review the COGARCH($p,\,q$) model proposed in Brockwell et al. (2006) and present some results on risk quantification in the univariate framework. In Sect. 3 we construct an ICA-COGARCH(1, 1) model for the joint dynamics of log prices and used it in a portfolio selection problem. In Sect. 4 we present some empirical results. Section 5 concludes the paper.

## COGARCH($p, \,q$) model

Let $L=(L_t)_{t\ge 0}$ be a pure jump Lévy process with finite variation. We define $(G_t)_{t\ge 0}$ as a COGARCH($p,\,q$)process with $q \ge p$ if it satisfies the following system of stochastic differential equations:

1 $$\begin{aligned} \left\{ \begin{array}{l} \text{ d }G_t = \sqrt{V_t}\text{ d }L_t\\ V_t = a_0 + {\mathbf {a}}^\top Y_{t-}\\ \text{ d }Y_t = {\mathbf {B}}Y_{t-} \text{ d }t + \left( a_0 +{\mathbf {a}}^\top Y_{t-}\right) \text{ d }\left[ L,L\right] ^{\left( d\right) }_t \end{array} \right. \end{aligned}$$

where $Y_t \in {\mathbb {R}}^{q}$ is a state vector process defined as:

$$\begin{aligned} Y_t = \left[ Y_{1,t}, \ldots , Y_{q,t}\right] ^{\top }, \end{aligned}$$

matrix ${\mathbf {B}}\in {\mathbb {R}}^{q\times q}$ has the following form:

$$\begin{aligned} {\mathbf {B}} = \left[ \begin{array}{l@{\quad }l@{\quad }l@{\quad }l} 0 &{}\quad 1 &{}\quad \ldots &{}\quad 0 \\ \vdots &{}\quad \vdots &{}\quad \ddots &{}\quad \vdots \\ 0 &{}\quad 0 &{}\quad \ldots &{}\quad 1 \\ -b_q &{}\quad -b_{q-1} &{}\quad \ldots &{}\quad -b_1 \\ \end{array}\right] \end{aligned}$$

and ${\mathbf {a}} \in {\mathbb {R}}^{q}$ is vector defined as:

$$\begin{aligned} {\mathbf {a}} = \left[ a_1, \ldots , a_{p}, a_{p+1}, \ldots , a_{q} \right] ^{\top } \end{aligned}$$

with $a_{p+1}=\dots =a_{q}=0$; $\left[ L, L\right] ^{\left( d\right) }_t$ is the discrete part of the quadratic variation^3^ of the underlying Lévy process $(L_t)_{t\ge 0}$ and is defined as:

$$\begin{aligned} \left[ L, L\right] ^{\left( d\right) }_t=\sum _{0\le s\le t}\left( \varDelta L_s\right) ^2. \end{aligned}$$

It is worth to notice that the structure of a COGARCH($p,\,q$) model is similar to that of a GARCH($p,\,q$). Indeed, we have the same noise process $\left( L_t\right) _{t \ge 0}$ that drives the observable process $(G_t)_{t\ge 0}$ and the variance process $(V_t)_{t\ge 0}$. Furthermore, the variance process $(V_t)_{t\ge 0}$ is described through a Continuous ARMA (CARMA) model (see Brockwell and Davis 2016; Iacus and Mercuri 2015, for more details on CARMA models) driven by the quadratic variation of $(L_t)_{t\ge 0}$, while, in a GARCH(p, q) model, the variance is a discrete ARMA model driven by the squares of the innovations. As observed in Brockwell et al. (2006), the state space process $Y_t$ in (1) can be written as a stochastic recurrence equation as reported below:

$$\begin{aligned} Y_t = J_{s,t}Y_s+K_{s,t} \ \ s\le t, \end{aligned}$$

where for fixed *s* and *t*, $J_{s,t}$ is a random $q\times q$-matrix and $K_{s,t}$ is a random *q*-vector. Using the theory of stochastic recurrence equations, it is possible to establish sufficient conditions for the stationarity of a COGARCH(*p*, *q*) process, for the positivity of the variance process $(V_t)_{t\ge 0}$ and for the existence of higher-order unconditional moments of the process $(G_t)_{t\ge 0}$ (see Brockwell et al. 2006; Iacus et al. 2017, for details). Choosing $q=p=1$, the solution of system (1) coincides with the COGARCH(1, 1) proposed by Klüppelberg et al. (2004).

### Discrete-time approximation of COGARCH($p, \,q$)

We quickly review a result given in Iacus et al. (2018), particularly useful in the estimation of COGARCH($p,\,q$) models. If $(L_t)_{t\ge 0}$ is a finite variation process, it is possible to construct a sequence of discrete-time processes that converges to a COGARCH($p,\,q$) process in the Skorokhod distance^4^. For each *n*, we consider a sequence of natural numbers $\left( N_n\right) _{n \in {\mathbb {N}}}$ such that $\lim \limits _{n\rightarrow +\infty }N_n$ $=+\infty$ and identify a partition of the compact interval $[0,\,T]$ as follows:

2 $$\begin{aligned} 0=t_{0,n}\le t_{1,n} \le \cdots \le t_{i,n} \le \cdots \le t_{N_n, n}. \end{aligned}$$

On the partition in (2), it is possible to define recursively the discrete process $G_{i,n}$ as:

3 $$\begin{aligned} G_{i,n} = G_{i-1,n}+\sqrt{V_{i-1,n}\varDelta t_{i,n}}\epsilon _{i,n} \end{aligned}$$

with

4 $$\begin{aligned} V_{i,n} = a_{0}+{\mathbf {a}}^{\top }Y_{i,n} \end{aligned}$$

where the innovations $(\epsilon _{i,n})_{n\in {\mathbb {N}}}$ are constructed using the first jump approximation method introduced in Maller and Szimayer (2007) (see Appendix A for details). By construction, $(\epsilon _{i,n})_{n\in {\mathbb {N}}}$ have zero mean and unitary variance. The discrete state process $Y_{i,n}$ is given by:

5 $$\begin{aligned} Y_{i,n}&=C_{i,n}Y_{i-1,n}+D_{i,n} \nonumber \\ C_{i,n}&=\left( I+\epsilon _{i,n}^{2}\varDelta t_{i,n}\mathbf {ea}^{\top }\right) e^{{\mathbb {B}}\varDelta t_{i,n}} \nonumber \\ D_{i,n}&=a_{0}\epsilon _{i,n}^{2}\varDelta t_{i,n}{\mathbf {e}}. \end{aligned}$$

As shown in Iacus et al. (2018), the pair^5^$(G_{i,n},V_{i,n})_{n\in {\mathbb {N}}}$ converges in the Skorokhod distance to the solution $\left( G_{t},V_{t}\right) _{t \ge 0}$ of the system in (1). We are particularly interested in the behavior of $(G_{i,n})_{n\in {\mathbb {N}}}$ and $(V_{i,n})_{n\in {\mathbb {N}}}$ for $p=q=1$ where the random coefficients $C_{i,n}$, $D_{i,n}$ and the variance process $V_{i,n}$ read:

6 $$\begin{aligned} C_{i,n}&=\left( 1+\epsilon _{i,n}^{2}\varDelta t_{i,n}a_{1}\right) e^{-b_{1}\varDelta t_{i,n}} \nonumber \\ D_{i,n}&=a_{0}\epsilon _{i,n}^{2}\varDelta t_{i,n} \nonumber \\ V_{i,n}&= a_{0}+a_{1}Y_{i,n}. \end{aligned}$$

Observing that:

$$\begin{aligned} Y_{i,n} = \frac{V_{i,n}-a_0}{a_1}=\left( 1+\epsilon ^{2}_{i,n}\varDelta t_{i,n}a_1\right) e^{-b_1\varDelta t_{i,n}}\frac{V_{i-1,n}-a_0}{a_1} +a_0\epsilon ^2_{i,n}\varDelta t_{i,n}, \end{aligned}$$

by straightforward algebra, we get:

7 $$\begin{aligned} \begin{aligned} V_{i,n}&= \;a_0\left( 1-e^{-b_1\varDelta t_{i,n}}\right) +\varDelta t_{i,n}a_1e^{-b_1\varDelta t_{i,n}}\epsilon ^{2}_{i,n}V_{i-1,n}+e^{-b_1\varDelta t_{i,n}}V_{i-1,n}\\&\quad + a_1a_0\epsilon ^2_{i,n}\varDelta t_{i,n}\left( 1-e^{-b\varDelta t_{i,n}}\right) . \end{aligned} \end{aligned}$$

Notice that since $\epsilon ^{2}_{i,n}\varDelta t_{i,n}V_{i-1,n}=\left( G_{i,n}-G_{i-1,n}\right) ^2$, the dynamics in (7) is similar to that of the variance in a GARCH(1, 1) process with an additional term $a_1a_0\epsilon ^2_{i,n}\varDelta t_{i,n}(1-$ $e^{-b_1\varDelta t_{i,n}})$ that depends on the i.i.d. innovations $\epsilon _{i,n}$. If we consider the Taylor expansion of $1-e^{-x}$ and apply it to the terms $1-e^{b_1\varDelta t_{i,n}}$ in (7), we get:

8 $$\begin{aligned} V_{i,n}=a_0b_1\varDelta t_{i,n}+\varDelta t_{i,n}a_1e^{-b_1\varDelta t_{i,n}}\epsilon ^{2}_{i,n}V_{i-1,n}+e^{-b_1\varDelta t_{i,n}}V_{i-1,n}+o\left( \varDelta t_{i,n}\right) . \end{aligned}$$

We recall that by construction $\epsilon _{i,n}$ has zero mean and unitary variance; thus, the process identified by $G_{i,n}$ in (3) with $V_{i,n}$ in (8) can be well approximated with a GARCH(1, 1) for small values of $\varDelta t_{n}:=\max _{i=1,\ldots N_n} \varDelta t_{i,n}$. More precisely, if we consider an equally spaced grid, i.e., $\forall i, \ \varDelta t_{i,n}=\frac{T}{N_n}=\varDelta t_{n}$ we have:

$$\begin{aligned} G_{i,n}&= G_{i-1,n}+\sigma _{i,n}\epsilon _{i,n} \\ \sigma ^2_{i+1,n}&= \omega _{0,n}+\alpha _{n}\sigma ^2_{i,n}\epsilon ^2_{i,n}+\beta _{n} \sigma ^2_{i,n} \end{aligned}$$

where

9 $$\begin{aligned} {\left\{ \begin{array}{ll} \sigma _{i,n} &{}:= \sqrt{V_{i-1,n}\,\varDelta t_{n}} \\ \omega _{0,n} &{}:= a_0b_1\varDelta t^2_{n} \\ \beta _{n} &{}:= e^{-b_1\varDelta t_{n}} \\ \alpha _n &{}:= a_1\varDelta t_n. \end{array}\right. } \end{aligned}$$

The reparameterization in (9) allows to obtain the GARCH(1, 1) specification as a special case of the COGARCH(1, 1) in the case of equally spaced time grids. The discrete process in (5) has been used in Iacus et al. (2018) for the construction of a pseudo-maximum likelihood estimation procedure for a COGARCH(*p*, *q*) model based on the assumption of normality for $\epsilon _{i,n}$. This procedure generalizes the approach proposed in Maller et al. (2008) for a COGARCH(1, 1) model. In Appendix B we provide some details on the pseudo-maximum likelihood method for the estimation of COGARCH($p,\,q$) models.

Once we have the COGARCH($p,\,q$) parameters, we can estimate the increments of the underlying Lévy $\widehat{\varDelta L_t}$ using the approach introduced in Iacus et al. (2017) based on the explicit solution of the state process $Y_t$ in (1). The estimated increments $\widehat{\varDelta L_t}$ allow us to obtain the distribution of process $G=(G_t)_{t\ge 0}$ for any time *t* using the bootstrap methodology Carlstein (1986).

### Risk measures in a COGARCH($p, \,q$) model

Let $(\varOmega ,{\mathcal {F}}, ({\mathcal {F}}_t)_{t\ge 0}, {\mathbb {P}})$ be a filtered probability space. We suppose the price of a risky asset with initial value $P_{0}$ to be modeled as:

10 $$\begin{aligned} P_{t}=P_{0}\exp (\mu t +G_t) \end{aligned}$$

where $\mu$ is a real constant and $G=(G_t)_{t\ge 0}$ is a COGARCH($p,\,q$) described through the relations in (1). Using (10), we define a loss function ${\mathcal {L}}_t$ as:

11 $$\begin{aligned} {\mathcal {L}}_t = P_0\left[ 1-\exp \left( \mu t +G_t\right) \right] . \end{aligned}$$

Consequently, $\textsf {VaR}_{\alpha }\left( {\mathcal {L}}_t\right)$ and $\textsf {ES}_{\alpha }\left( {\mathcal {L}}_t\right)$ are computed, respectively, as:

12 $$\begin{aligned} \textsf {VaR}_{\alpha }\left( {\mathcal {L}}_t\right)&:=\inf \left( l: \ F_{{\mathcal {L}}_t}\left( l\right) \ge 1-\alpha \right) \nonumber \\ \textsf {ES}_{\alpha }\left( {\mathcal {L}}_t\right)&:=E\left( {\mathcal {L}}_t\left| {\mathcal {L}}_t \ge \textsf {VaR}_{\alpha }\left( {\mathcal {L}}_t\right) \right. \right) \end{aligned}$$

where $F_{{\mathcal {L}}_t}\left( l\right) =\textsf {Pr}\left( {\mathcal {L}}_t\le l\right)$ is the cumulative distribution function (cdf) of the loss at time *t*. The two risk measures in (12) depend only on the distribution of the loss function ${\mathcal {L}}_t$ at a fixed horizon *t*. In our framework, under the assumption that the series $\ln \left( P_t\right)$ is ergodic and observed on an irregular spaced grid, the loss distribution can be obtained following these steps:

- Let $P_0, P_1,\ldots ,P_i,\ldots ,P_n$ be the prices observed at $t_0, t_1,\ldots , t_i, \ldots ,t_n$. The drift $\mu$ is estimated as follows:
  $$\begin{aligned} {\hat{\mu }}=\frac{\sum _{i}^n X_{i}}{n} \qquad \text {where} \qquad X_i =\frac{\ln \left( P_i\right) -\ln \left( P_{i-1}\right) }{\varDelta t_i} \end{aligned}$$
  and $\varDelta t_i=t_i-t_{i-1}$. Starting from:
  $$\begin{aligned} X_i = \mu +\frac{G_{i}-G_{i-1}}{\varDelta t_i} \end{aligned}$$
  the estimator ${\hat{\mu }}$ is written as^6^:
  13 $$\begin{aligned} {\hat{\mu }}=\mu +\frac{1}{n}\sum _{i=1}^{n}\frac{G_i-G_{i-1}}{\varDelta t_i}. \end{aligned}$$
- Estimate the discrete-time sequence ${\widehat{G}}_0,{\widehat{G}}_1,\ldots ,{\widehat{G}}_i,\ldots ,{\widehat{G}}_n$ obtained from (10).
- Apply the maximum pseudo-likelihood approach in Iacus et al. (2018) in order to obtain the COGARCH(($p,\,q$) parameters. The underlying Lévy process is estimated used the first jump approximation scheme. We provide some details in Appendix B.
- Estimated Lévy increments and COGARCH parameters are used as inputs for the distribution of loss function ${\mathcal {L}}_t$ at any maturity *t*. Simulated values are obtained through block bootstrapping (see Carlstein (1986) for details). Finally, compute $\textsf {VaR}_{\alpha }\left( {\mathcal {L}}_t\right)$ and $\textsf {ES}_{\alpha }\left( {\mathcal {L}}_t\right)$ in (12) using their sample estimators.

## ICA-COGARCH ($p, \,q$) model and portfolio selection

We consider a one period $[0,\,T]$ portfolio optimization problem where the investor is not allowed to rebalance the portfolio in an intermediate time instant $t \in [0,\,T]$. We consider an optimization problem where the objective function is a linear combination of the expected value of the final gain $\mathcal {G_{T}}$ and the risk associated with the final loss ${\mathcal {L}}_T$ where ${\mathcal {L}}_T=-{\mathcal {G}}_T$.

The considered market is composed of ${\bar{N}}$ risky assets whose prices are the entries of the ${\bar{N}}$-dimensional stochastic process $P_{t}=\left[ P_{1,t},\ldots .P_{{\bar{N}},t}\right]$. We denote with $c_{i}$ the number of shares of the *i*-th asset bought at time 0. The static optimization problem can be formalized as follows:

14 $$\begin{aligned} \begin{array}{c} \underset{c_{1},\ldots c_{{\bar{N}}}}{\max }U\left( {\mathcal {L}}_T\right) :=-E\left[ {\mathcal {L}}_T\right] -\lambda \rho \left( {\mathcal {L}}_T\right) \\ \text {s.t.} \left\{ \begin{array}{l} {\sum }_{i=1}^{c_{{\bar{N}}}}c_{i}P_{i,0}=W_0\\ c_{i} \ge 0 \quad \forall \, i=1,\ldots c_{{\bar{N}}} \\ \end{array} \right. \\ \end{array} \end{aligned}$$

The final loss ${\mathcal {L}}_T$ is defined as:

15 $$\begin{aligned} {\mathcal {L}}_T = W_0-W_T = \sum _{i=1}^{{\bar{N}}}c_{i}\left( P_{i,0}-P_{i,t}\right) \end{aligned}$$

where $W_0$ and $W_T$ are the initial and the final wealth, respectively; $\lambda$ is a positive constant representing the marginal contribution of the risk measure to the objective function $U\left( {\mathcal {L}}_T\right)$, i.e., an additional unit of the risk measure $\rho \left( {\mathcal {L}}_T\right)$ corresponds to a reduction of $\lambda$ units in the objective function $U\left( {\mathcal {L}}_T\right)$; intuitively^7^$\lambda$ can be also interpreted as the risk aversion. In our framework short selling is not allowed. If the risk measure $\rho \left( {\mathcal {L}}_T\right)$ is a positive homogeneous function, the objective function in (14) can be rewritten as:

16 $$\begin{aligned} U({\mathcal {L}}_T) = W_0\left[ \sum _{i=1}^{{\bar{N}}}\frac{c_i P_{i,0}}{W_0}E\left( \frac{P_{i,t}}{P_{i,0}}-1\right) -\lambda \rho \left( \sum _{i=1}^{{\bar{N}}}\frac{c_i P_{i,0}}{W_0}\left( 1-\frac{P_{i,t}}{P_{i,0}}\right) \right) \right] . \end{aligned}$$

Let $R_{i,t}=\frac{P_{i,t}}{P_{i,0}}-1$ denote the linear return on the interval $\left[ 0,T\right]$. Defining $w_{i}=\frac{c_i P_{i,0}}{W_0}$ as the portfolio weight, the problem in (14) can be written equivalently as:

17 $$\begin{aligned} \begin{array}{c} W_0\left[ \underset{w_{1},\ldots w_{{\bar{N}}}}{\max }{\sum }_{i=1}^{{\bar{N}}}w_{i}E\left( R_{i,t}\right) -\lambda \rho \left( -{\sum }_{i=1}^{{\bar{N}}}w_i R_{i,t}\right) \right] \\ \text {s.t.} \left\{ \begin{array}{l} {\sum }_{i=1}^{{\bar{N}}}w_i=1\\ w_{i} \ge 0 \quad \forall \, i=1,\ldots {\bar{N}}\\ \end{array} \right. \\ \end{array} \end{aligned}$$

Observe that the solution $w^{\star }=\left[ w_1^{\star },\ldots ,w_{{\bar{N}}}^{\star }\right] ^{\top }$ of (17) does not depend on the initial wealth $W_0$. For the computation of expected returns, only the marginal distributions are required, while for the risk measure computed on the loss distribution of the portfolio we need the joint distribution of assets. In Sect. 3.1 we review the mathematical description of the ICA. In Sect. 3.2 we present the ICA-COGARCH($p,\,q$) model for portfolio selection. Section 3.3 provides some theoretical results in terms of convergence.

### Independent Component Analysis

The Independent Component Analysis (ICA) introduced in Comon (1994) is a statistical method that considers observed series as a linear transformation of latent independent signals. Under the assumption that the observed return series are linear combinations of unobserved independent components, the observed vector process $X=\left[ X_{1},\ldots ,X_{{\bar{N}}}\right] ^\top$ can be expressed as:

18 $$\begin{aligned} X = A \,\, S, \end{aligned}$$

where *A* is a ${\bar{N}}\times K$ mixing matrix and the vector *S* contains *K* independent random variables called independent components (ICs). The independent components are determined by looking for a $K\times {\bar{N}}$ matrix *W* such that:

19 $$\begin{aligned} S = W \,\, X. \end{aligned}$$

There are different statistical methodologies for obtaining the independent components in *S* and the linear transformation matrix *W* based for example on maximum likelihood or maximization of any non-Gaussianity measure. In particular, the Fast Fixed Point algorithm FastICA proposed in Hyvarinen and Oja (1997) is an iterative algorithm based on the maximization of the negentropy, a measure of non-Gaussianity. The Independent Component Analysis is a statistical method to analyze data that allow us to work on a linear transformation of observed time series with the advantage of dealing with independence.

### Portfolio selection using ICA-COGARCH($p, \,q$) model

The ICA-COGARCH($p,\,q$) model combines the COGARCH($p,\,q$) discussed in Sect. 2 with the ICA. The idea is similar to that developed in the ICA-GARCH($p,\,q$) model (see Wu and Philip 2005, for details), but we have to adapt it to a context of irregular time grids. Given a set of irregularly spaced time series, the starting point is the definition of a common grid. The points of the grid can be obtained as the union of time points for each asset quotation where for missing data we can use linear interpolation; this grid still contains irregularities due to the daily market closing, weekends and holidays. In a context with standard activity for all assets in the portfolio, we expect the final grid to resemble the grid of the most liquid asset. An alternative approach could be that of discarding points with missing data for at least one asset. In this framework, we expect the final grid to be similar to the grid of the less liquid asset. Although this second approach does not require the use of any imputation method since we do not have missing data, it seems not to be reasonable to apply it in the presence of highly illiquid assets as it would neglect a lot of information.

In the proposed model, we use the ICA algorithm to recover the independent signals and then model each signal as a COGARCH($p,\,q$). Indeed, we consider a market composed of ${\bar{N}}$ assets with prices where the dynamics of the *i*-th asset price is:

20 $$\begin{aligned} P_{i,t}=P_{i,0}\exp \left[ \mu _{i}t+X_{i,t}\right] \ \ i=1,\ldots ,{\bar{N}}, \end{aligned}$$

where $\mu _{i} \in {\mathbb {R}}$, while the vector process $X_{t}:=\left[ X_{1,t}\ldots ,X_{1,{\bar{N}}}\right] ^{\top }$ is described through the ICA-COGARCH ($p,\,q$) model defined as:

21 $$\begin{aligned} X_{t}=A\,\,S_{t}, \end{aligned}$$

*A* is a ${\bar{N}}\times K$ matrix and $S_{t}=\left( S_{1,t},\ldots ,S_{K,t}\right) ^\top$ is a *K*-vector process where each entry $S_{i,t}$ is a COGARCH($p,\,q$) model defined in (1).The optimization problem in (17) requires the joint distribution of the linear returns $R_{T}=\left[ R_{1,T},\ldots ,R_{{\bar{N}},T}\right] ^\top$ that can be simulated using the following steps:

- From the observed data estimate the mixing matrix *W* in (19), the COGARCH($p,\,q$) parameters for each independent component $S_{i,t}$ and the increments of underlying Lévy process.
- Using the estimated increments, simulate *M* sample paths of independent signals $\left( S_{1,t},\ldots ,S_{K,t}\right)$ until maturity *T*.
- Using the simulated signals $\left( S_{1,t},\ldots ,S_{K,t}\right)$, generate the corresponding sample path of asset prices $P_{1,t},\ldots P_{{\bar{N}},t}$ for $t\in \left[ 0,T\right]$.
- Compute the linear returns $R_{T}=\left[ R_{1,t},\ldots ,R_{{\bar{N}},T}\right] ^\top$ on the simulated prices.

The next step is the approximation of the objective function in (17). We consider the sample estimators for the expected return and for the risk measure (VaR or ES).

### Convergence of the approximated process to the ICA-COGARCH($p,\,q$) model

Using the same arguments in Iacus et al. (2018) we construct a piecewise constant vector process $X_{t}^{n}=AS_{t}^{n}$ defined on the partition of the interval $[0,\,T]$ introduced in (2). The piecewise vector process $S^{n}_t$ has the following form:

22 $$\begin{aligned} S^{n}_{t}=\left[ S^{n}_{t,1},\ldots ,S^{n}_{t,{\bar{N}}}\right] ^{\top }, \end{aligned}$$

where for any $j\in \left\{ 1,\ldots ,{\bar{N}}\right\}$, the process $S^{n}_{t,j}$ is:

$$\begin{aligned} S^{n}_{t,j} := S^{n}_{i,j}, \text { with } t\in \left[ t_{i-1,n},t_{i,n}\right) , \end{aligned}$$

$S^{n}_{i,j}$ has the same dynamics described in (3). Here we will prove the uniform convergence in probability of the process $X_{t}^{n}$ to $X_t$ as $n\rightarrow +\infty$. Let $\left\| \cdot \right\|$ and $\left\| \cdot \right\| _M$ be the Euclidean norm and its induced matrix norm, respectively, the following inequality holds:

23 $$\begin{aligned} \left\| X_{t}^{n}-X_{t}\right\|= & {} \left\| AS_{t}^{n}-AS_{t}\right\| \nonumber \\= & {} \left\| A\left( S_{t}^{n}-S_{t}\right) \right\| \nonumber \\\le & {} \left\| A\right\| _M\left\| S_{t}^{n}-S_{t}\right\| \end{aligned}$$

where $\left\| A\right\| _M:={\sup }_{\left\| x\right\| \ne 0}\frac{\left\| Ax\right\| }{\left\| x\right\| }$. As shown in Iacus et al. (2018), each component of the vector $S_t^{n}$ converges uniformly in probability to $S_t$; therefore, we have:

$$\begin{aligned} \underset{0\le t\le T}{\sup }\left\| X^{n}_t-X_t\right\|\le & {} \left\| A\right\| _M \underset{0\le t\le T}{\sup } \left\| S^{n}_t-S_t\right\| \\\le & {} \left\| A\right\| _M \underset{0\le t\le T}{\sup } \sum _{j=1}^{{\bar{N}}}\left| S_{t,j}^n-S_t\right| \\\le & {} \left\| A\right\| _M \sum _{j=1}^{{\bar{N}}} \underset{0\le t\le T}{\sup } \left| S_{t,j}^n-S_t\right| , \end{aligned}$$

that implies the uniform convergence in probability of $X_{t}^{n}$ to $X_{t}$ as $n\rightarrow +\infty$.

**Fig. 1:**
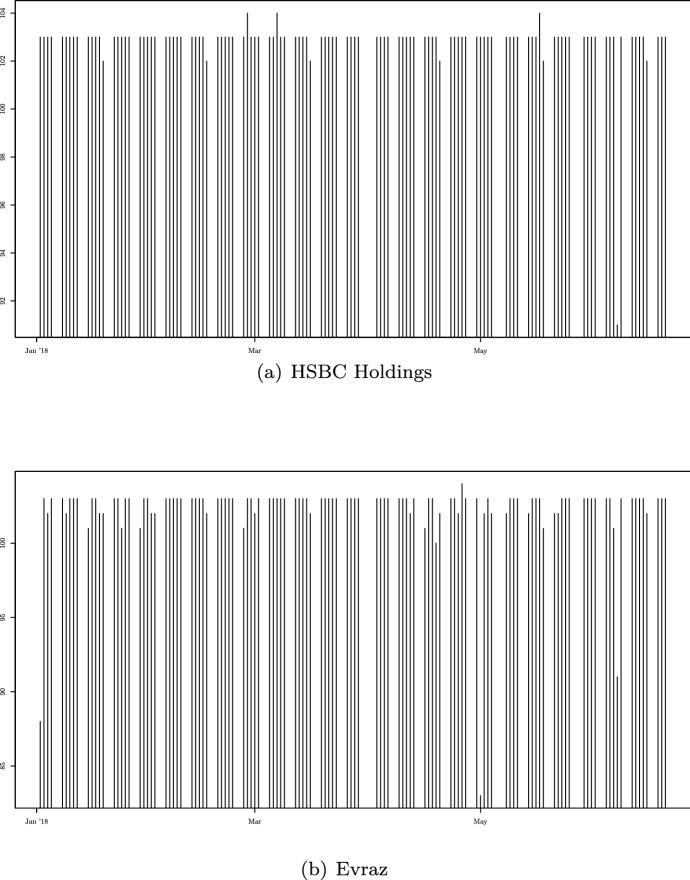
Daily number of observed quotations for the most capitalized asset (upper plot) and the least capitalized asset (lower plot) of the FTSE 100 Index in 2018

## Empirical analysis

We consider two high-frequency (HFT) datasets composed of prices of the components in the FTSE 100 Index in 2018 and 2020. Intraday prices are collected every five minutes^8^ from the beginning of January 2018 at market opening (09:00 AM) to mid-July 2018 at market closing (05:30 PM) and from the end of February 2020 to the beginning of September 2020 following the same opening-closure time pattern. The datasets are selected to study the performance of the proposed model during relatively stable market conditions (as observed in the dataset referring to the calendar year 2018) and during a period of severe financial stress (observed in the first half of 2020)^9^.

The analysis performed revolves around two main parts. We first determine optimal weights using the methodology described in Sect. 3.2 with an in-sample window of length one month^10^. Then, we study the out-of-sample portfolio performance in terms of monetary wealth for different lengths of the out-of-sample window (one week, two weeks and three weeks), i.e., the rebalancing period^11^.

Figure 1 reports the number of daily quotations for the most and the least capitalized members of the FTSE 100 Index at the beginning of 2018 from where we can have an idea if irregularities in the time grids of quoted prices.

**Table 1:** Members of the FTSE 100 Index with the corresponding weights (in %) in 2018

Constituent	Index weight	Constituent	Index weight	Constituent	Index weight
Anglo American	0.90%	Hargreaves Lansdown	0.24%	Rio Tinto	2.31%
Associated British Food	0.48%	Halma	0.25%	Royal Mail	0.27%
Admiral Group	0.20%	HSBC Holdings	7.34%	Rightmove	0.30%
Ashtead Group	0.53%	Int. Consolidated Airlines	0.55%	Rolls-Royce Holdings	0.79%
Antofagasta	0.18%	InterContinental Hotels Group	0.45%	Randgold Resources	0.30%
Aviva	1.09%	3i	0.45%	RSA Insurance Group	0.35%
AstraZeneca	3.41%	Imperial Brands	1.27%	Rentokil Initial	0.27%
BAE Systems	1.02%	Informa	0.32%	Sainsbury (J)	0.21%
Barclays	1.93%	Intertek Group	0.41%	Schroders	0.20%
British American Tobacco	5.20%	ITV	0.30%	Sage Group	0.38%
Barratt Developments	0.29%	Just Eat	0.22%	Segro	0.33%
Berkeley Group Holdings	0.26%	Johnson Matthey	0.32%	Shire	1.75%
British Land Co	0.35%	Kingfisher	0.35%	Smurfit Kappa Group	0.38%
BHP Billiton	1.61%	Land Securities Group	0.37%	SKY	0.74%
Bunzl	0.39%	Legal & General Group	0.84%	Standard Life Aberdeen	0.54%
BP	5.08%	Lloyds Banking Group	2.54%	DS Smith	0.26%
Burberry Group	0.39%	London Stock Exchange Group	0.71%	Smiths Group	0.33%
BT Group	1.05%	Micro Focus International	0.23%	Scottish Mortgage Investment	0.34%
Coca-Cola HBC AG	0.28%	Marks & Spencer Group	0.24%	Smith & Nephew	0.64%
Carnival	0.44%	Mondi Group	0.39%	SSE	0.72%
Centrica	0.44%	Melrose Industries	0.13%	Standard Chartered Bank	1.06%
Compass Group	1.26%	Morrison (Wm) Supermarkets	0.25%	St James’s Place	0.31%
Croda International	0.32%	National Grid PLC	1.50%	Severn Trent	0.24%
CRH	1.11%	NMC Health	0.15%	Tesco	1.09%
DCC	0.32%	Next	0.36%	TUI	0.37%
Diageo	3.22%	Ocado Group	0.63%	Taylor Wimpey	0.33%
Direct Line Insurance Group	0.29%	Paddy Power Betfair	0.33%	Unilever	2.51%
Evraz	0.12%	Prudential	2.53%	Utilities Group	0.27%
Experian	0.77%	Persimmon	0.43%	Vodafone Group	2.85%
easyJet	0.22%	Pearson	0.32%	WPP	0.76%
Ferguson	0.73%	Reckitt Benckiser Group	2.07%	Whitbread	0.37%
Fresnillo	0.13%	Royal Bank of Scotland Group	0.46%		
Glencore	2.27%	Royal Dutch Shell A	5.65%		
GlaxoSmithKline	3.71%	Royal Dutch Shell B	4.70%		
GVC Holdings	0.29%	RELX	0.85%		

The first dataset is composed of 14078 points of successive time instants where we have price quotations for at least one asset in the portfolio. We have 7927 points in the original grid with at least one missing data for the components of the FTSE 100 Index^12^. Following the strategy of removing the whole row in the dataset with at least one missing data, we obtain a loss of information due to the elimination of 55% of the original observations; the final dataset would contain only 621,251 observations. Moreover, the maximum distance $\varDelta t$ between two subsequent trading instants within a trading day would become 180 minutes instead of the 10 minutes observed in the original grid. The alternative approach, based on the numerical approximation on the dataset of prices, introduces only 2% of artificial data. Analyzing the frequency of the missing data in each point in the time grid, we observe that we have only one missing data in 3799 of the cases and two missing data in 2096 of the cases. To complete the dataset, we use linear interpolation since we have no more than two missing data in 74.35% of the cases with at least one missing point in the trading day.

The second dataset is composed of 14086 points of successive time instants: we have 29110 missing data over the total 1422686 points (almost 2%) and we have 5705 time instants with at least one missing data for the components. If we follow the strategy of removing the whole row that contains a missing data we have a loss of about 40% of the original observations; the dataset of 2020 is composed of 846,481 observations with a similar behavior of the first dataset in terms of the maximum distance (160 min) within a trading day. Again we introduce use linear interpolation to complete the dataset.

In both cases, the final grid is still irregular and reflects the grid of most liquid components of the FTSE 100 Index.

We first check whether the ICA algorithm based on the maximization of the negentropy measure is able to extract components that are at least approximately independent. We base our analysis on the mutual information of the two random variables *X* and *Y* (see Comon 1994, for a complete discussion about the mutual information in the ICA) defined as:

$$\begin{aligned} I\left( X,Y\right) =\sum _{i}\sum _{j} p\left( x_i,y_j\right) \ln \left[ \frac{p\left( x_i,y_j\right) }{p\left( x_i\right) p\left( y_j\right) }\right] . \end{aligned}$$

Observe that $I\left( X,Y\right)$ is equal to zero if two random variables are independent.

Table 2 contains the main statistics of the pairwise mutual information of extracted components for each in sample window during 2018, while Table 3 refers to the same quantities but on the components extracted from the second dataset, i.e., data of 2020. All quantities are computed using the R package infotheo Meyer (2014).

**Table 2:** Pairwise mutual information of independent components for each in sample window (length 30 days) from January 2018 to July 2018

2018 windows	Min.	1st Qu.	Median	Mean	3rd Qu.	Max.
1st in-sample	2.576e–02	4.256e–02	4.764e–02	5.029e–02	5.380e–02	2.923e–01
2nd in-sample	9.150e–03	1.883e–02	2.246e–02	2.463e–02	2.719e–02	1.965e–01
3rd in-sample	2.171e–02	3.769e–02	4.270e–02	4.510e–02	4.872e–02	2.712e–01
4th in-sample	2.132e–02	3.728e–02	4.244e–02	4.495e–02	4.833e–02	2.428e–01
5th in-sample	2.307e–02	3.873e–02	4.413e–02	4.664e–02	4.996e–02	3.841e–01
6th in-sample	2.372e–02	3.870e–02	4.358e–02	4.625e–02	4.930e–02	2.934e–01
7th in-sample	2.161e–02	3.721e–02	4.190e–02	4.433e–02	4.753e–02	2.585e–01
8th in-sample	1.766e–02	3.150e–02	3.615e–02	3.860e–02	4.182e–02	2.771e–01
9th in-sample	2.344e–02	4.058e–02	4.597e–02	4.810e–02	5.207e–02	2.366e–01
10th in-sample	2.338e–02	4.245e–02	4.751e–02	5.012e–02	5.383e–02	2.604e–01

**Table 3:** Pairwise mutual information of independent components for each in sample window (length 30 days) from February 2020 to September 2020

2020 windows	Min.	1st Qu.	Median	Mean	3rd Qu.	Max.
1st in-sample	1.453e–02	2.960e–02	3.462e–02	3.652e–02	4.047e–02	2.198e–01
2nd in-sample	1.746e–02	3.083e–02	3.566e–02	3.781e–02	4.120e–02	2.383e–01
3rd in-sample	2.472e–02	4.189e–02	4.703e–02	4.928e–02	5.300e–02	2.573e–01
4th in-sample	2.164e–02	3.515e–02	3.975e–02	4.218e–02	4.538e–02	2.248e–01
5th in-sample	2.483e–02	4.198e–02	4.719e–02	4.968e–02	5.336e–02	2.600e–01
6th in-sample	2.776e–02	4.601e–02	5.118e–02	5.370e–02	5.745e–02	2.450e–01
7th in-sample	2.441e–02	4.293e–02	4.828e–02	5.053e–02	5.425e–02	2.572e–01
8th in-sample	7.081e–03	1.947e–02	2.297e–02	2.511e–02	2.723e–02	1.824e–01
9th in-sample	3.197e–02	4.811e–02	5.369e–02	5.624e–02	6.010e–02	2.514e–01
10th in-sample	2.335e–02	3.784e–02	4.261e–02	4.529e–02	4.839e–02	2.547e–01
11th in-sample	2.919e–02	4.724e–02	5.285e–02	5.535e–02	5.921e–02	2.787e–01
12th in-sample	1.912e–02	3.330e–02	3.796e–02	4.021e–02	4.323e–02	2.252e–01

**Table 4:** Mean and standard errors of the Modified Herfindahl Index in the considered out-of-sample windows of 2018 where RP stands for rebalancing period

2018 - MH		$\rho \left( {\mathcal {L}}\right) ={{\mathsf {VaR}}}_{5\%}$		$\rho \left( {\mathcal {L}}\right) ={{\mathsf {ES}}}_{5\%}$	
$\lambda$	RP	Mean	SD	Mean	SD
2	2 weeks	4.518e–04	1.326e–04	4.656e–04	1.064e–04
2	3 weeks	5.630e–04	2.615e–04	4.950e–04	1.509e–04
5	2 weeks	3.738e–04	8.322e–05	4.214e–04	2.095e–04
5	3 weeks	4.064e–04	8.222e–05	5.077e–04	2.903e–04
10	2 weeks	3.851e–04	9.869e–05	4.272e–04	2.496e–04
10	3 weeks	4.345e–04	1.432e–04	4.931e–04	1.825e–04
15	2 weeks	1.759e–02	8.362e–04	1.740e–02	6.123e–04
15	3 weeks	4.518e–04	1.326e–04	4.656e–04	1.064e–04

**Table 5:** Mean and standard errors of the Modified Herfindahl Index in the considered out-of-sample windows of 2020 where RP stands for rebalancing period

2020 - MH		$\rho \left( {\mathcal {L}}\right) ={\mathsf {VaR}}_{5\%}$		$\rho \left( {\mathcal {L}}\right) ={\mathsf {ES}}_{5\%}$	
$\lambda$	RP	Mean	SD	Mean	SD
2	2 weeks	1.137e–03	2.063e–03	8.126e–04	6.416e–04
2	3 weeks	9.528e–04	5.137e–04	1.065e–03	3.882e–04
5	2 weeks	1.058e–03	1.465e–03	8.807e–04	6.705e–04
5	3 weeks	9.131e–04	5.551e–04	8.391e–04	4.775e–04
10	2 weeks	1.014e–03	1.475e–03	6.898e–04	5.134e–04
10	3 weeks	8.199e–04	4.160e–04	7.725e–04	4.245e–04
15	2 weeks	8.281e–04	9.913e–04	6.143e–04	2.737e–04
15	3 weeks	9.602e–04	5.986e–04	9.157e–04	6.476e–04

It is worth noting that, in both datasets, mutual information is close to zero for each in sample window denoting a situation relatively close to independence for almost all components. In all windows we do not observe pairs of extracted components with mutual information larger than 0.4; the average value for the mutual information is of the order of $10^{-2}$.

We proceed with our analysis by fitting a COGARCH(1, 1) model to the independent components^13^. We present results on portfolio optimization for different values of $\lambda$ (we consider $\lambda =\left( 2, 5, 10, 15\right)$) and two different out-of-sample windows, respectively, 2 and 3 weeks.

We discuss the stability of portfolio weights using two measures, namely the Modified Herfindahl Index $H_{1,t}$ and the portfolio Turnover Index $\tau _t$, respectively, defined as:

$$\begin{aligned} H_{1,t}:=\frac{\sum _{i=1}^{{\bar{N}}} w_i\left( t\right) ^2-\frac{1}{{\bar{N}}}}{1-\frac{1}{{\bar{N}}}}, \ \tau _t:=\sum _{i=1}^{{\bar{N}}}\left| w_{i}\left( t\right) -w_{i}\left( t-1\right) \right| . \end{aligned}$$

By construction, the Modified Herfindahl Index ranges from zero to one: the lower bound corresponds to the equally weighted portfolio, while the upper bound to the case where the whole wealth is invested in a single asset. The Modified Herfindahl Index gives an intuition on the level of diversification/concentration of the portfolio for a given strategy. From Table 4, we observe for the first dataset that the strategies based on the Value at Risk seem to be produce results closer to the equally weighted portfolio than the corresponding strategies based on the Expected Shortfall. The lowest value for the Herfindahl index is obtained for $\lambda =5$ and 2-week out-of-sample windows with ${{\mathsf {VaR}}}$ as a risk measure, while the highest value is obtained for $\lambda =15$ and 2-week out-of sample windows with ${{\mathsf {ES}}}$ in the objective function.

Table 5 contains information on the Modified Herfindahl Index of portfolios built using data of 2020. Results suggest that portfolio weights are not to far from the equally weighted strategy. The smallest value for the index is obtained using the portfolio selection strategy based on ${{\mathsf {VaR}}}_{5\%}$ with $\lambda =10$ and 3-week out-of-sample window, while the strategy based on ${{\mathsf {ES}}}_{5\%}$ displays the lowest value for the Modified Herfindahl Index is obtained with $\lambda =15$ and 2-week out-of-sample window.

The portfolio Turnover Index $\tau _{t}$ can be seen as a proxy of the transaction costs as the composition of portfolio weights changes at time *t*. A value close to zero denotes a stability of the weights. From Table 6 we observe that the values of the Turnover Index in the first dataset are always lower than 0.21 that suggests a stability of weights when ${\mathsf {VaR}}$ or ${\mathsf {ES}}$ are considered as risk measures in our optimization problem. For the second dataset, the values of the Turnover Index in Table 7 seem to assume larger values in line with the greater perceived uncertainty during the COVID 19 crisis. In the second dataset, the situation seems to be slightly different in this as the maximum value is more than 0.3.

**Table 6:** Mean and standard errors of the Turnover Index in the considered out-of-sample windows of 2018 where RP stands for rebalancing period

2018 - TI		$\rho \left( {\mathcal {L}}\right) ={\mathsf {VaR}}_{5\%}$		$\rho \left( {\mathcal {L}}\right) ={\mathsf {ES}}_{5\%}$	
$\lambda$	RP	Mean	SD	Mean	SD
2	2 weeks	1.779e–01	1.925e–02	1.945e–01	2.674e–02
2	3 weeks	2.095e–01	3.056e–02	1.880e–01	2.893e–02
5	2 weeks	1.387e–01	2.414e–02	1.420e–01	3.718e–02
5	3 weeks	1.724e–01	1.698e–02	1.911e–01	5.225e–02
10	2 weeks	1.432e–01	2.584e–02	1.463e–01	3.802e–02
10	3 weeks	1.755e–01	1.920e–02	1.882e–01	3.501e–02
15	2 weeks	7.260e–02	7.670e–03	7.401e–02	1.287e–02
15	3 weeks	1.779e–01	1.925e–02	1.945e–01	2.674e–02

**Table 7:** Mean and standard errors of the Turnover Index in the considered out-of-sample windows of 2020 where RP stands for rebalancing period

2020 - TI		$\rho \left( {\mathcal {L}}\right) ={\mathsf {VaR}}_{5\%}$		$\rho \left( {\mathcal {L}}\right) ={\mathsf {ES}}_{5\%}$	
$\lambda$	RP	Mean	SD	Mean	SD
2	2 weeks	2.788e–01	2.394e–01	2.434e–01	1.533e–01
2	3 weeks	2.801e–01	1.118e–01	3.017e–01	5.492e–02
5	2 weeks	2.822e–01	2.020e–01	2.701e–01	1.333e–01
5	3 weeks	2.518e–01	9.604e–02	2.492e–01	1.003e–01
10	2 weeks	2.633e–01	2.120e–01	2.178e–01	1.367e–01
10	3 weeks	2.396e–01	7.631e–02	2.314e–01	8.210e–02
15	2 weeks	2.347e–01	1.595e–01	1.996e–01	9.488e–02
15	3 weeks	2.732e–01	1.172e–01	2.551e–01	1.078e–01

**Fig. 2:**
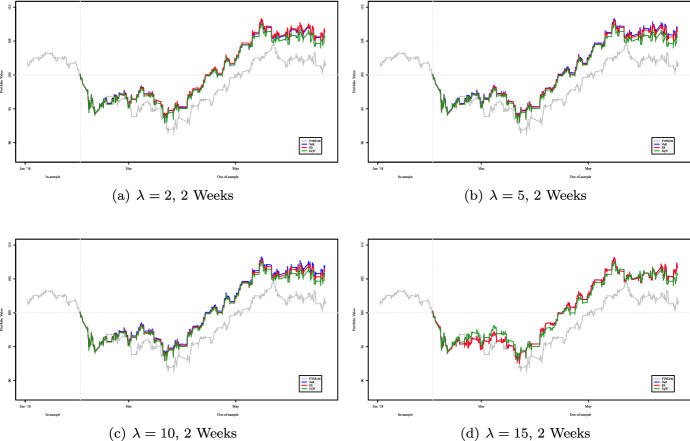
Out-of-sample performance of portfolios obtained using two-week rebalancing period for varying values of $\lambda$ for the period January 2018–July 2018

**Fig. 3:**
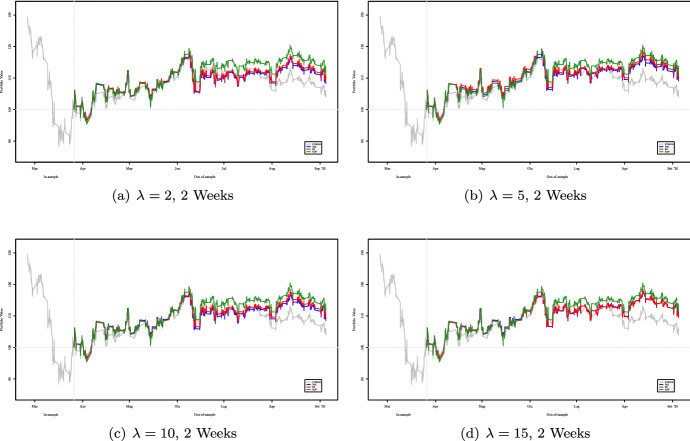
Out-of-sample performance of portfolios obtained using two-week rebalancing period for varying values of $\lambda$ for the period January 2020–July 2020

**Fig. 4:**
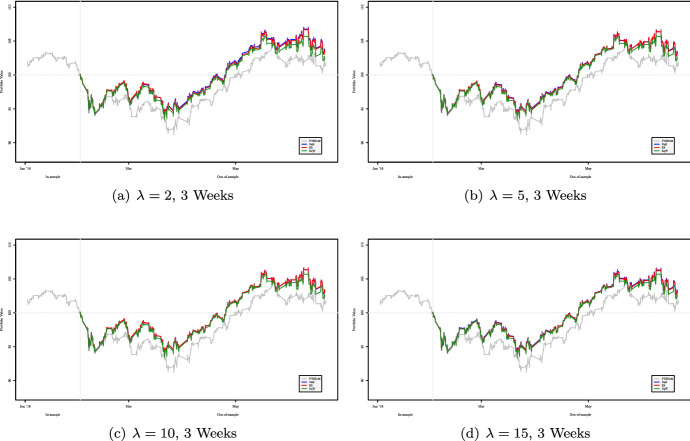
Out-of-sample performance of portfolios obtained using three-week rebalancing period for varying values of $\lambda$ for the period January 2018–July 2018

**Fig. 5:**
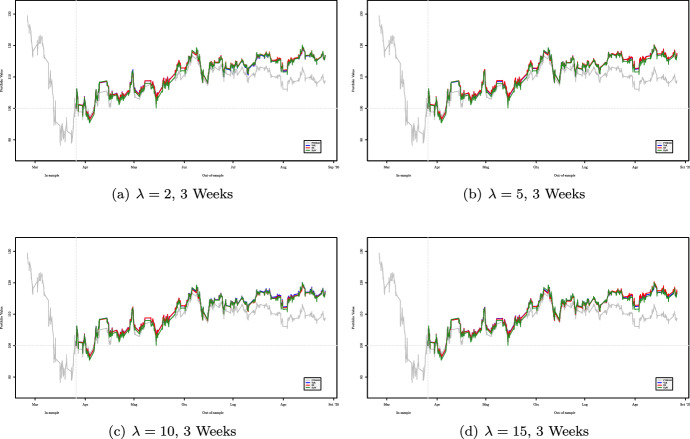
Out-of-sample performance of portfolios obtained using three-week rebalancing period for varying values of $\lambda$ for the period February 2020–September 2020

Figures 2, 3, 4 and 5 show the cumulative performance in terms of monetary wealth, respectively, for two- and three-week out-of-sample windows. In the first dataset the two strategies, i.e., ${\mathsf {VaR}}$ and ${\mathsf {ES}}$ based, consistently outperform the FTSE 100 Index and most of the time perform better than the equally weighted portfolio.

To investigate further the out-of-sample results, we consider the maximum drawdown index ${\mathsf {MaxDD}}$ associated with the wealth $W_t$ over $\left[ 0,T\right]$ defined as:

$$\begin{aligned} {\mathsf {MaxDD}}=\underset{\tau \in \left( 0,T\right) }{\max }\left[ 0,\underset{t\in \left( 0,\tau \right) }{\max } \left( W_t-W_{\tau }\right) \right] . \end{aligned}$$

This measure allows to have a first intuition of the maximum loss for a given portfolio strategy. The ${\mathsf {MaxDD}}$ index is particularly convenient in our framework since it can be easily computed in a context with irregular time grids.

Based on the results of the first dataset presented in Tables 8, 9 and 10 we can affirm that our approach provides better results in terms of the Maximum Drawdown, for the ${\mathsf {VaR}}$ and ${\mathsf {ES}}$-based strategies, than those obtained using the equally weighted approach or the strategy of investing the entire sum on the FTSE 100 Index. In particular, the approach based on VaR seems to be the most conservative^14^.

**Table 8:** Out-of-sample Maximum Drawdown of portfolios obtained using two-week rebalancing period for varying values of $\lambda$ for the period January 2018–July 2018

**2018**	$\lambda =2$	$\lambda =5$	$\lambda =10$	$\lambda =15$
Index	8.423e–02	8.423e–02	8.423e–02	8.423e–02
VaR	7.119e–02	**7.061e–02**	**7.025e–02**	7.891e–02
ES	**7.087e–02**	7.153e–02	7.197e–02	7.901e–02
Eqw	7.336e–02	7.336e–02	7.336e–02	**7.336e–02**

**Table 9:** Out-of-sample Maximum Drawdown of portfolios obtained using two-week rebalancing period for varying values $\lambda$ for the period February 2020–September 2020

**2020**	$\lambda =2$	$\lambda =5$	$\lambda =10$	$\lambda =15$
Index	3.257e–01	3.257e–01	3.257e–01	3.257e–01
VaR	1.094e–01	**1.050e–01**	1.046e–01	**9.989e–02**
ES	1.091e–01	1.055e–01	**1.008e–01**	1.025e–01
Eqw	**1.080e–01**	1.080e–01	1.080e–01	1.080e–01

Tables 9 and 11 refer to the Maximum Drawdown of, respectively, for 2-week and 3-week rebalancing periods for the second dataset. Except for the case $\lambda =2$ with out-of-sample windows of length 2 weeks, the portfolio strategies based on ${\mathsf {VaR}}$ and ${\mathsf {ES}}$ perform better than the equally weighted strategy.

**Table 10:** Out-of-sample Maximum Drawdown of portfolios obtained using three-week rebalancing period for varying values of $\lambda$ for the period January 2018–July 2018

**2018**	$\lambda =2$	$\lambda =5$	$\lambda =10$	$\lambda =15$
Index	8.423e–02	8.423e–02	8.423e–02	8.423e–02
VaR	**6.458e–02**	**6.484e–02**	6.608e–02	**6.568e–02**
ES	6.544e–02	6.590e–02	**6.586e–02**	6.648e–02
Eqw	6.825e–02	6.825e–02	6.825e–02	6.825e–02

**Table 11:** Out-of-sample Maximum Drawdown of portfolios obtained using three-week rebalancing period for varying values of $\lambda$ for the period February 2020–September 2020

**2020**	$\lambda =2$	$\lambda =5$	$\lambda =10$	$\lambda =15$
Index	3.257e–01	3.257e–01	3.257e–01	3.257e–01
VaR	9.790e–02	**9.852e–02**	9.763e–02	**9.902e–02**
ES	**9.542e–02**	9.952e–02	**9.870e–02**	9.906e–02
Eqw	1.079e–01	1.079e–01	1.079e–01	1.079e–01

Table 12 reports the excess performance with respect to the equally weighted strategy for different levels of transaction costs. We can observe that the transaction costs seem not to influence the ranking of models in almost all cases where the strategies based on the ICA-COGARCH model outperform the equally weighted portfolio in the original analysis (i.e., with no transaction costs). Indeed we have only three cases where the inclusion of transaction costs leads to a change of sign in the final performance. This fact seems to be coherent with the small values of the Turnover Index.

**Table 12:** Out-of-sample excess performance of the proposed approach using as benchmark the equally weighted strategy for different levels of transaction costs expressed in basis points (bps)

Strategy	$\lambda$	RP	2018			2020		
			No costs	5bps	10bps	No costs	5bps	10bps
VaR	2	2 weeks	0.7512	0.7297	-0.0430	-3.1199	-3.2702	-3.4202
ES	2	2 weeks	0.9082	0.8866	0.8650	-2.5796	-3.2481	-3.3761
VaR	2	3 weeks	1.1135	1.0975	1.0815	0.0530	-0.0410	-0.1348
ES	2	3 weeks	0.9526	0.9365	0.9204	0.0056	-0.0497	-0.1523
VaR	5	2 weeks	1.1841	1.1626	1.1410	-2.2121	-2.3652	-2.5182
ES	5	2 weeks	0.8669	0.8456	0.8241	-1.4752	-2.3560	-2.4998
VaR	5	3 weeks	0.7968	0.7809	0.7649	0.6659	0.5804	0.4949
ES	5	3 weeks	0.7161	0.7001	0.6841	0.6735	0.5809	0.4959
VaR	10	2 weeks	1.0735	1.0520	1.0305	-2.7653	-2.9094	-3.0532
ES	10	2 weeks	0.5709	0.5496	0.5282	-2.0507	-2.8816	-2.9977
VaR	10	3 weeks	0.6849	0.6690	0.6532	0.5225	0.4412	0.3600
ES	10	3 weeks	0.7414	0.7254	0.7094	0.5254	0.3682	0.2191
VaR	15	2 weeks	1.1042	1.0836	1.0630	-1.7548	-1.8848	-2.0145
ES	15	2 weeks	1.0929	1.0724	1.0517	-1.8265	-1.8605	-1.9660
VaR	15	3 weeks	0.6732	0.6572	0.6413	0.3820	0.2893	0.1967
ES	15	3 weeks	0.5579	0.5419	0.5260	0.8932	0.2957	0.2094

## Conclusion

In this study, we use continuous-time models for the dynamics of the independent components extracted from real market time series. The independence of the components and the estimation algorithm for COGARCH($p,\,q$) models proposed in Iacus et al. (2018) constitutes the main ingredients of a portfolio optimization problem where the objective function is expressed as a linear combination of expected portfolio wealth and a homogeneous risk measure. Through an empirical analysis, performed on the members of the FTSE100 Index, we observe that the out-of-sample performance of the portfolio in the proposed framework does not depend on the specific risk measure (VaR or ES) but seems to be sensitive to $\lambda$ coefficient.

The advantage of our approach is found in the formulation of a mathematical problem for portfolio selection that incorporates information generated both from price level and time distribution of market quotations. Compared to recent machine learning techniques that look for patterns of asset dynamics useful in price prediction, we have a direct interpretation of model parameters that allows to perform a sensitivity analysis.

## Figures and Tables

**Fig. 6:**
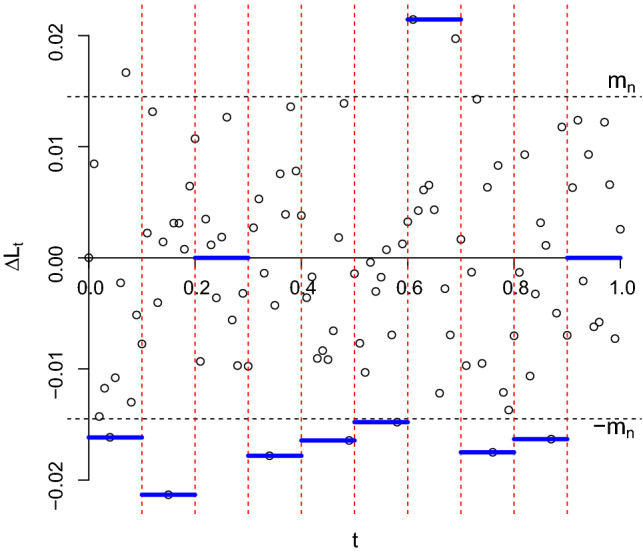
First jump approximation of a Lévy process $L_t$ based on the stopping times $\tau _{i,n}$ defined as in (24) where the magnitude of the increments exceeds the barrier $m_n$. The blue line refers to the trajectory of $\mathbb {1}_{\tau _{i,n}<\infty }\varDelta L_{\tau _{i,n}}$

## A First jump approximation of a Lévy process

A pure jump Lévy process $\left( L_t\right) _{t\ge 0}$ is identified by the triplet $\left( \gamma ,0,\pi \left( x\right) \right)$ where $\gamma$ is a real scalar, while $\pi \left( x\right)$ is a Lévy measure with characteristic function written as:

$$\begin{aligned} E\left( e^{i\theta L_t}\right) =\exp \left[ i\theta \gamma t+ t\int _{{\mathbb {R}}\backslash 0}\left( e^{i\theta x}-1-i\theta x \mathbb {1}_{\left| x\right| \le 1}\right) \pi \left( \text{ d }x\right) \right] , \ t\ge 0. \end{aligned}$$

For each $n\in {\mathbb {N}}$, we consider a sequence of natural numbers $N_n$ such that $\lim \nolimits _{n\rightarrow +\infty }N_n=+\infty$ to define the following partition of the interval $\left[ 0,T\right]$:

$$\begin{aligned} 0=t_{0,n}\le t_{1,n}\le \cdots \le t_{N_n,n}=T. \end{aligned}$$

Let $\varDelta t_n={\max }_{i=1,\ldots N_n}t_{i,n}-t_{i-1,n}$ with the requirement $\varDelta t_n\rightarrow 0$ as $n\rightarrow +\infty$. We introduce the sequence of positive scalars $m_n$ such that $m_n\le 1$ and $m_n\rightarrow 0$ as $n\rightarrow +\infty$.If the Lévy measure $\pi \left( x\right)$ satisfies the condition:

$$\begin{aligned} \underset{n\rightarrow +\infty }{\lim } \varDelta t_n {\bar{\varPi }}^2\left( m_n\right) =0 \end{aligned}$$

with ${\bar{\varPi }}\left( x\right) =\int _{\left| y\right| >x}\pi \left( \text{ d }y\right)$, we can construct a sequence of stopping time processes $\tau _{i,n}$ defined as:

24 $$\begin{aligned} \tau _{i,n}=\left\{ \begin{array}{ll} \inf \left( t\in \left[ t_{i-1,n},t_{i,n}\right) : \left| \varDelta L_t\right| >m_n\right) &{} \text {if there exists at least an increment}\\ &{} \text {with a magnitude greater than } m_n \\ &{} \text { in the interval } \left[ t_{i-1,n},t_{i,n}\right) \\ &{} \\ +\infty &{} \text {otherwise} \\ \end{array} \right. \end{aligned}$$

with $\varDelta L_t=L_{t}-L_{t-}$. For each *n*, we associate to the stopping time process a sequence of independent random variables $\left( \mathbb {1}_{\tau _{i,n}<\infty }\varDelta L_{\tau _{i,n}}\right) _{i=1,\ldots ,N_n}$. As shown in Fig. 6, the random variable $\mathbb {1}_{\tau _{i,n}<\infty }\varDelta L_{\tau _{i,n}}$ refers to the interval $\left[ t_{i-1,n},t_{i,n}\right)$ and corresponds to the first jump of $L_t$ in the *i*-th interval whose magnitude exceeds $m_n$ if such a jump occurs and zero otherwise (Fig. 6).

Under the requirement $E\left[ L_1^2\right] <+\infty$, we construct the innovations $\left( \epsilon _{i,n}\right) _{i=1,\ldots ,N_n}$ as:

$$\begin{aligned} \epsilon _{i,n}=\frac{\mathbb {1}_{\tau _{i,n}<\infty }\varDelta L_{\tau _{i,n}}-v_{i,n}}{\eta _{i,n}} \end{aligned}$$

where $v_{i,n}$ and $\eta _{i,n}$ are, respectively, the mean and the standard error of the random variable $\mathbb {1}_{\tau _{i,n}<\infty }\varDelta L_{\tau _{i,n}}$.

## B pseudo-maximum likelihood estimation procedure of a COGARCH($p, \,q$) process

Let $\left( \varOmega ,{\mathcal {F}}, ({\mathcal {F}}_t)_{t\ge 0}, {\mathbb {P}}\right)$ be a filtered probability space. We observe the following quantities $G_{t_0},G_{t_1},\ldots ,G_{t_i},\ldots ,G_{t_N}$ and assume they are the realizations of a COGARCH(p,q) model as in (1), i.e., we write the increments as follows:

$$\begin{aligned} \varDelta G_{t_i}=G_{t_i}-G_{t_{i-1}}=\int _{t_{i-1}}^{t_i} \sqrt{V_u}\text{ d }L_u. \end{aligned}$$

The likelihood of the increments has the following form:

$$\begin{aligned} {\mathcal {L}}\left( a_0,{\mathbf {a}},{\mathbf {B}}\right)= & {} f\left( \varDelta G_{t_N},\varDelta G_{t_{N-1}},\ldots ,\varDelta G_{t_{i}},\ldots ,\varDelta G_{t_{1}}\right) \\= & {} \prod _{i=1}^N f\left( \varDelta G_{t_{i}}\left| {\mathcal {F}}_{t_{i-1}}\right. \right) . \end{aligned}$$

In general the conditional density $f\left( \varDelta G_{t_{i}}\left| {\mathcal {F}}_{t_{i-1}}\right. \right)$ is unknown. In the pseudo-maximum likelihood approach we can assume this conditional density to be a Gaussian with mean $E\left[ \varDelta G_{t_{i}}\left| {\mathcal {F}}_{t_{i-1}}\right. \right] =0$ and variance (see Brockwell et al. 2006; Iacus et al. 2017, for more details on the calculation of the first two moments of a COGARCH(p,q) model):

25 $$\begin{aligned} Var\left[ \varDelta G_{t_{i}}\left| {\mathcal {F}}_{t_{i-1}}\right. \right] = m_2 \frac{a_0 b_q \varDelta t_i }{b_q-a_1 m_2}+m_2{\mathbf {a}}^{\top }\left[ e^{{\tilde{B}}\varDelta t_i}{\tilde{B}}^{-1}\left( I-e^{-{\tilde{B}}\varDelta t_i}\right) \right] \left( Y_{t_{i-1}}-E\left( Y_{\infty }\right) \right) \end{aligned}$$

where $m_2=\int x^2 \pi \left( \text{ d }x\right)$ is the second moment of the Lévy measure $\pi$; $E\left( Y_{\infty }\right)$ is the first unconditional moment of the state process $Y_t$ in (1) and ${\tilde{B}}={\mathbf {B}}+m_2 {\mathbf {e}}{\mathbf {a}}^{\top }$. The substitution of the real density with the normal one is widely applied in the estimation of stochastic differential equations, see for instance Yoshida (1992) where the driving noise is a Brownian Motion and recently Masuda (2013); Masuda and Uehara (2017) for the case where the driving noise is an ergodic Lévy process.

Denoting with $\hat{{\mathcal {L}}}\left( a_0,{\mathbf {a}},{\mathbf {B}}\right)$ the pseudo-likelihood, i.e., the likelihood under the normality assumption, we need to filter from the dataset a proxy for the unobservable state process $Y_{t}$ necessary for the computation of the variance in (25). Starting from the discretization in (5), we write the dynamics of the discrete-time process $Y_{t_i}$ as follows:

26 $$\begin{aligned} Y_{t_{i}}=\left( I+\epsilon _{i}^{2}\varDelta t_{i}\mathbf {ea}^{\top }\right) e^{{\mathbf {B}}\varDelta t_{i}}Y_{t_{i-1}}+\alpha _{0}\epsilon _{i}^{2}\varDelta t_{i}{\mathbf {e}}. \end{aligned}$$

From (3) and (4), we get

27 $$\begin{aligned} \varDelta t_{i} \epsilon _i^2=\frac{\left( G_{t_{i}}-G_{t_{i-1}}\right) ^2}{a_0+{\mathbf {a}}^{\top }Y_{t_{i-1}}}. \end{aligned}$$

Substituting (27) into (26), we obtain:

28 $$\begin{aligned} Y_{t_{i}}=\left( I+\frac{\left( G_{t_{i}}-G_{t_{i-1}}\right) ^2}{a_0+{\mathbf {a}}^{\top }Y_{t_{i-1}}}\mathbf {ea}^{\top }\right) e^{{\mathbf {B}}\varDelta t_{i}}Y_{t_{i-1}}+\alpha _{0}\frac{\left( G_{t_{i}}-G_{t_{i-1}}\right) ^2}{a_0+{\mathbf {a}}^{\top }Y_{t_{i-1}}}{\mathbf {e}}. \end{aligned}$$

It is worth noting that, given a set of model parameters and a initial value $Y_{t_0}$, we can univocally determine the sequence $Y_{t_1},Y_{t_2},\ldots , Y_{t_{N-1}}$ through the relation in (28) and the observed increments $\varDelta G_{t_i}$. Therefore we can compute the conditional variance in (25) and evaluate the pseudo-likelihood $\hat{{\mathcal {L}}}\left( a_0,{\mathbf {a}},{\mathbf {B}}\right)$ function. We estimate the model parameters solving the following maximization problem:

$$\begin{aligned} \left( a_0^{\star },{\mathbf {a}}^{\star },{\mathbf {B}}^{\star }\right) =\underset{a_0,{\mathbf {a}},{\mathbf {B}} \in \varTheta }{\text {argmax}} \ \hat{{\mathcal {L}}}\left( a_0,{\mathbf {a}},{\mathbf {B}}\right) , \end{aligned}$$

where the set $\varTheta$ is composed of the model parameters that ensure stationarity, existence of the mean for the state process $Y_t$ and non-negativity of the variance process $V_t$ (see Brockwell et al. 2006, for more details).

From the estimated parameters $\left( a_0^{\star },{\mathbf {a}}^{\star },{\mathbf {B}}^{\star }\right)$ we are able to obtain get an estimator $Y^{\star }_{t_i}$ based on the dynamics in 26. The approximated underlying Lévy process ${\tilde{L}}^{\star }_t$ is obtained as follows:

$$\begin{aligned} {\tilde{L}}^{\star }_t=\sum _{t_i\le t}\frac{G_{t_i}-G_{t_{i-1}}}{a_0^{\star }+\left( {\mathbf {a}}^{\star }\right) ^{\top }Y^{\star }_{t_{i-1}}}. \end{aligned}$$

## Footnotes

1 This approach has been coined as the CHICAGO algorithm (Conditionally Heteroskedastic Independent Component Analysis of Generalized Orthogonal GARCH).

2 We consider the two risk measures Value at Risk (VaR) and Expected Shortfall (ES), but the framework is generic.

3 If $(L_t)_{t\ge 0}$ is a pure jump with finite variation, the quadratic variation is equal to its discrete part.

4 The Skorokhod distance between two processes *U*, *V* defined on $D^{d}\left[ 0,T\right]$, i.e., the space of càdlàg ${\mathbb {R}}^{d}$-valued stochastic processes on $[0,\,T]$, is:

$$\begin{aligned} \rho (U,V):=\underset{\lambda \in \varLambda }{\inf }\left\{ \underset{0\le t\le T}{\sup }\Vert U_{t}-V_{\lambda _t}\Vert +\underset{0\le t\le T}{\sup }|\lambda _t-t|\right\} \end{aligned}$$

where $\varLambda$ is a set of strictly increasing continuous functions with $\lambda _0=0$ and $\lambda _T=T$.

5 In order to avoid confusion, we do not use brackets and do not indicate time positivity in the discretized processes.

6 ${\hat{\mu }}$ is an unbiased estimator of $\mu$:

$$\begin{aligned} E\left[ {\hat{\mu }}\right] =\mu +\frac{1}{n} \sum _{i=1}^{n} \frac{E\left( G_{i}-G_{i-1}\right) }{\varDelta t_i} \end{aligned}$$

$E\left( G_{i}-G_{i-1}\right) =0$ when the underlying Lévy process $\left( L_t\right) _{t\ge 0}$ satisfies $E\left( L_1\right) =0$. The mean square error of the estimator ${\hat{\mu }}$ tends to zero as $n\rightarrow +\infty$. Indeed:

$$\begin{aligned} {\mathsf {MSE}}\left( {\hat{\mu }}\right)= & {} E\left[ \left( {\hat{\mu }}-\mu \right) ^2\right] = \frac{1}{n^2}E\left[ \left( \sum _{i=1}^{n} \frac{G_{i}-G_{i-1}}{\varDelta t_i}\right) ^2\right] \\= & {} \frac{1}{n^2}\sum _{i=1}^{n}\frac{E\left[ \left( G_{i}-G_{i-1}\right) ^2\right] }{\left( \varDelta t_i\right) ^2} +\frac{2}{n^2}\sum _{i=1}^{n-1} \sum _{j=i+1}^{n}\frac{E\left[ \left( G_{i}-G_{i-1}\right) \left( G_{j}-G_{j-1}\right) \right] }{\varDelta t_i\varDelta t_j}. \end{aligned}$$

Under the condition that ensures the existence of the second moment of the COGARCH process (see Brockwell et al. 2006, for more details) we have $E\left[ \left( G_{i}-G_{i-1}\right) \left( G_{j}-G_{j-1}\right) \right] =0$ and $\lim \nolimits _{n\rightarrow +\infty } {\mathsf {MSE}}\left( {\hat{\mu }}\right) =0$.

7 The formal proof of this intuition requires the axiomatic definition of the investor’s preferences.

8 Bloomberg provides price quotations with a minimum frequency of one minute. In order to minimize the impact of noise, we downsample the dataset to a five-minute frequency (see Hansen and Asger (2006) as reference).

9 The volatility of the FTSE100 Index in 2020, measured through the FTSE 100 IVI 30 index, almost doubles the values observed for the same index in 2018 with a peak that reaches the level 0.7.

10 For the first window we use the weights published by FTSE Russell as reported in Table 1.

11 The FTSE 100 Index is composed of 100 companies, but in Table 1 we observe 101 shares. This is due to the fact that Royal Dutch Shell has both class A and B shares listed. Both classes have identical rights, except for the dividend access mechanism which applies only to the class B of ordinary shares.

12 Having 101 shares, we observe 28717 missing data over the total 1,421,878 points that represent 2% of the sample size.

13 Fitted parameters for each window are available upon request.

14 It is worth to notice that ${\mathsf {MaxDD}}$ can be seen as the worst loss of a portfolio in a fixed time horizon.
